# Supplementary material for: Rapid, large-scale species discovery in hyperdiverse taxa using 1D MinION sequencing
Source: BMC Biol. 2019 Nov 29;17:96. doi: 10.1186/s12915-019-0706-9 (PMC6884855; doi:10.1186/s12915-019-0706-9)
Supplement: Supplementary file 1 — Additional file 1 : Figure S1. Pairwise edit distances between tags. Figure S2 and S3: Analyses of GenBank phorid data. [file 12915_2019_706_MOESM1_ESM.docx]

Additional File 1

Figure S1: Pairwise Levenshtein distances between designed tags.

Figure S2: Distribution of intraspecific pairwise distances for publically available barcodes in GenBank.

Figure S3: Match ratio between mOTUs at various thresholds and morphology for publically available barcodes from GenBank. Overall, highest match ratio is found for 3%.
